# Connecting the Micro to the Macro: An Exploration of Micro-Behaviors of Individuals Who Drive CSR Initiatives at the Macro-Level

**DOI:** 10.3389/fpsyg.2018.02417

**Published:** 2018-12-03

**Authors:** Latha Poonamallee, Simy Joy

**Affiliations:** ^1^The New School, New York, NY, United States; ^2^Norwich Business School, University of East Anglia, Norwich, United Kingdom

**Keywords:** CSR, positive psychology, organizational innovation, qualitative research, event structure analysis

## Abstract

Grounded on a case study on the formation of an inter-corporate CSR initiative in which four corporations from Chennai, India collaborate, this paper explores the micro-behaviors that individual actors engage in to create CSR solutions later adopted at the macro-organizational level. Based on the findings, the paper (1) identifies five categories of micro-behaviors, namely increasing stakeholder salience by turning attention to the ethical and social responsibilities to specific stakeholder groups, emerging as a self-appointed CSR champion by assuming personal responsibility for action, creating CSR initiative prototypes by leveraging personal skills, garnering support by leveraging personal networks and amassing operational resources by organizational resources; (2) explicates the characteristics of individual approach to CSR that makes it different from, but complementary to organizational approach to CSR.

## Introduction

The role of individual managers, executives, and owners in CSR and sustainability initiatives has recently begun to receive greater scholarly attention (Hemingway, 2005; Lee, 2008; Aguinis and Glavas, 2012; Ones et al., 2018). It is increasingly clear to both scholars and practitioners that behaviors at the individual level impact and shape a wide and complex range of macro level outcomes (Hemingway and Maclagan, 2004; Porter and Kramer, 2011; Amit and Zott, 2012; Littlewood, 2014; Ones et al., 2018). Individual actors often act as self-appointed change agents (Walley and Stubbs, 1999; Walley, 2000) unafraid to abandon the commercial rhetoric and take the first steps down toward truly socially responsible actions on their organization's behalf acting as “corporate entrepreneurs” (Hemingway, 2005) making small experiments to bring about a fairer world (Longsdon and Wood, 2002). Research provides examples of insiders initiating and championing transformative initiatives such as sustainability (Ones et al., 2018), and community level capacity building and economic self-sufficiency (Duarte, 2010).

An important area of inquiry is how individual actions contribute to organizational level CSR actions and outcomes (Bhattacharya et al., 2009). Research on the links between individual actions at the micro level and organizational CSR at the macro level, is still an emerging area (Griffin and Prakash, 2014; Ghobadian et al., 2015). Although few exceptions exist (Ones et al., 2018), most research explores and explains the effects of CSR on employees (Brammer et al., 2015; West et al., 2015) and not as often about how individual actions may shape organizational CSR priorities and processes.

This paper is a contribution to the growing body of literature addressing this gap. We present an analysis of case events and individual actions that led to the creation of an inter-organizational CSR initiative in India targeted at under-privileged urban youth, and identify and describe the micro-actions of individual managers and executives that eventually surface as CSR initiatives of their organizations. Based on the findings, we compare and contrast individual and corporate CSR approaches and discuss their mutual differences as well as complementarities.

## Whither the individual?

Examining the role of individual actions in advancing CSR is a recently emerging area of research (Hemingway and Maclagan, 2004; Hemingway, 2005; Augilera et al., 2007; Aguinis and Glavas, 2012; Glavas, 2016; Ones et al., 2018). Understanding individuals' role in CSR requires answering four key questions: *Who are the individuals driving CSR? What drives them? How do individuals shape CSR initiatives? How is the individual approach to CSR different from organizational approach to CSR?* Although notable exceptions exist (Ones and Dilchert, 2012; Ones et al., 2018; Wiernik et al., 2018), a large proportion of current empirical literature on individual role in CSR largely focuses on the “*who” and what'* questions and not the “*how” questions*.

### The who and what

Hemingway (2005, p. 238) offers the term “Corporate Social Entrepreneur” to refer to individuals “who operate within the corporation in a socially entrepreneurial manner and is motivated by a social, as opposed to other agenda.” Substantial amount of empirical research has considered how top-level actors such as CEOs and Top Management Teams influence CSR (e.g., Godos-Diez et al., 2011), while others examine actors occupying lower positions in the hierarchy as originators of CSR and sustainability behaviors (Ones and Dilchert, 2012; Ones et al., 2018; Wiernik et al., 2018).

This line of research is grounded in applying what we know about human psychology and motivation and how it affects behaviors in terms of individual level decisions, actions, and outcomes. Scholars have addressed this question by identifying different and sometimes co-existing and competing motives and mental models that may drive individual actors (Poonamallee and Goltz, 2014). For instance, Augilera et al. (2007, p. 839) put forward three different motives—“*instrumental* (self-interest driven), *relational* (concerned with the relationships among the group members) and *moral* (concerned with ethical standards and moral principles).” Empirical studies show that other-oriented values originating from moral motives have a greater impact on CSR (Desai and Rittenburg, 1997; Graafland et al., 2007). For example, companies demonstrate higher levels of CSR when top managers score high in community orientation (Lerner and Fryxell, 1994) and a CEO's compassion for others and community orientation correlate with higher levels of CSR engagement by his or her company (Agle and Caldwell, 1999). Managerial values such as benevolence and integrity act as a driver for social change (Hemingway and Maclagan, 2004; Andersson et al., 2007; Choi and Wang, 2007).

### The how

Researchers have documented a number of models of organization-driven CSR models (for a review, see Maon et al., 2009), but since their focus is on the macro level, they do not address micro-level actions. Moreover, most of them describe organization-driven CSR as impersonal and rational process, following a top-down approach closely tied to corporate strategy. The common steps include assessment of stakeholder salience and needs in order to determine the scope of CSR action required, establishing its link with corporate strategy, formulation of CSR goals, policies, and guidelines, design and implementation of programs, benchmarking and evaluation, and publicizing the outcomes within and outside of the organization (Khoo and Tan, 2002; Werre, 2003; Cramer et al., 2004; Maon et al., 2009). Even though it is argued that individual and corporate moral responsibility influence and enhance each other (Constantinescu and Kaptein, 2015), most CSR models assume individuals who occupy roles designated for the purpose carry out organizational-level processes they function as objects and vehicles of the corporate goal, rather than subjects and moral agents. These models do not address how the involvement of individual actors feeds into the organizational processes or result in creation of CSR.

Although, a few scholars have suggested that individual actors exert their influence on CSR through day-to-day micro-actions while participating in various organizational processes (Augilera et al., 2007; Maon et al., 2009; Duarte, 2010; Slack et al., 2015), empirical research that systematically explores the micro-actions of individuals and how they drive CSR is still emergent. When individuals are considered in empirical CSR studies, it is mostly to study how macro level CSR/CR programs impact micro-level outcomes such as employee commitment (West et al., 2015), employees' creative effort (Brammer et al., 2015), employee relations and psychological contracts (Rayton et al., 2015). The unilateral and collaborative CSR/CR mechanisms identified by Griffin and Prakash (2014) are unidirectional flowing from the corporation to community, employee or other stakeholder group and not about how individuals affect and shape organizational action. This lack of integration of individual level actions into organizational-level CSR process models is a significant gap in literature.

If individuals, not organizations, are moral actors (Hemingway and Maclagan, 2004), and if what lies behind seemingly rational organizational processes are the interactions, sense making, and decision-making by individuals (Takkala and Pallab, 2000; Duarte, 2010), it is necessary to acknowledge and understand the micro-processes of individuals and how they add to macro-processes and outcomes. This study seeks to uncover micro-behaviors of individuals involved in creation of what later emerges as CSR initiatives of their organizations. In doing this, we capture the personal, *ad-hoc*, emergent and iterative nature of individual approaches to CSR as opposed to the impersonal, rational, strategic and planned nature of organization-driven CSR as depicted in the prevalent models (Maon et al., 2009).

### Research method

This paper is uses a case study approach (Yin, 2018) as it allows for inductive theory building (Eisenhardt, 1989). The case traces the formation of Livelihood Advancement Business School (LABS) in Chennai, India, that was set up as an inter-organizational CSR partnership of four corporations, for the purpose of providing underprivileged urban youth with life and employment skills. The specific focus is on delineating the involvement and actions of the four key actors in shaping up this initiative.

### Data sources

Both primary and secondary data were collected. Primary data consisted of audio-taped individual and, focus group interviews and observational data. Interviews were approximately an hour long and were transcribed for coding and thematic analysis using NVivo software. A total of 25 individual interviews were conducted. This included all the 4 key actors who were instrumental in the formation of LABS Chennai, and all of the 5 facilitators/teachers employed at LABS-Chennai, 5 alumni and 11 students. These individual interviews were supplemented by five focus group interviews with all the students of the LABS Chennai center. Students and alumni are beneficiaries of the initiative and while they provide contextual information, the focus was on the 4 key actors who were instrumental in formation of LABS-Chennai. Secondary data included reports, newsletters, and news reports from the print and online media.

### Data analysis

We took an iterative analytic approach to the data, which could be roughly classified into three phases. In the first phase, we wrote a thick description of the case combining the data from multiple sources. The purpose was to capture in detail all the events and actions involved in the formation of LABS-Chennai.

In the second phase, we performed an Event Structure Analysis (ESA, Corsaro and Heise, 1990). ESA is a hybrid method (Green et al., 2015) that combines inductive, interpretive research with a positivistic explanatory mode of inquiry. ESA applied in case study scenarios allows the researchers to focus on both processes and outcomes helping develop a detailed description of underlying actions and mechanisms (Trumpy, 2008). ESA is facilitated by a computer program called ETHNO (Available at http://www.indiana.edu/~socpsy/ESA/). ETHNO while similar to NVivo as in it helps in analyzing qualitative data, ETHNO is also different because it facilitates the creation of replicable, causal sequences of an event sequence. Similar to grounded theory methodology, it allows the researcher to move through concrete to levels of abstraction but also forces the analyst to answer questions that clarify causal relationships and counterfactual explanations. This method has been successfully used in studies in various settings (Corsaro and Heise, 1990; Griffin, 1993; Uehara, 2001; O'Neill et al., 2007). For a more in-depth discussion, please refer to Corsaro and Heise (1990), Stevenson and Greenberg (1998) and Trumpy (2008).

**Figure 1:**
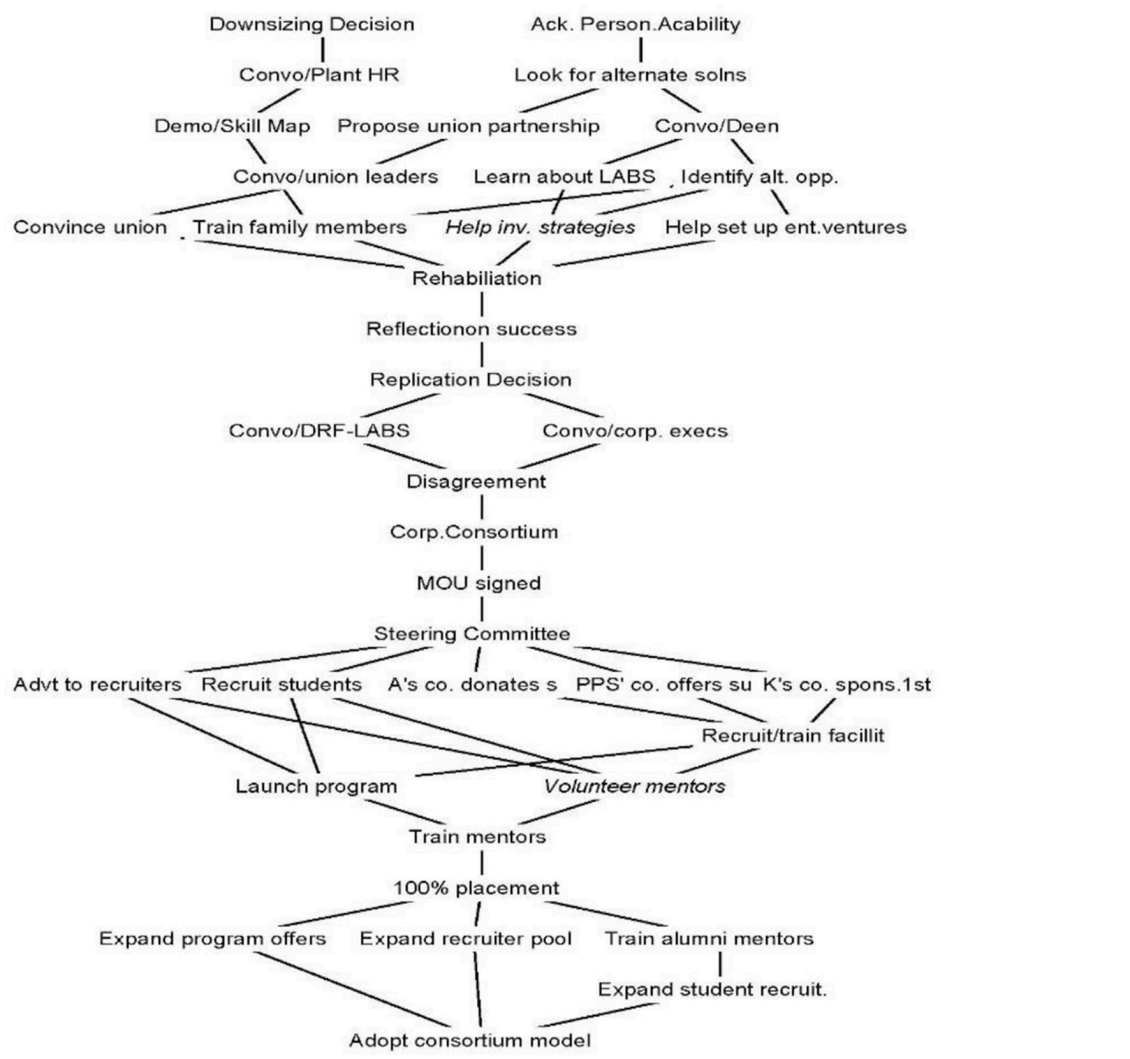
Events and Actions related formation of LABS-Chennai.

ETHNO uses the analyst's expert judgments (Griffin, 1993) and prompts the analyst to replace the temporal sequence with a causal logic (Griffin and Ragin, 1994). ETHNO uses a series of yes/no questions to clarify antecedents and four types of relationships between complex webs of events and actions*: historical causation, prerequisite, implications, and counterfactual relationships*. By alternating between these queries, one is able to clarify the causal relationships and identify key convergences (an event in which several actions converge) and divergences (an event that leads to multiple subsequent events and provides motivation and opportunities for action; Stevenson and Greenberg, 1998). As the first stage analysis, ETHNO creates a concrete event map, which in the second stage can be summarized into causal paths at the abstract level and in the third stage to cause and effect relations (For detailed steps, see Table 1). The Event Structure Analysis helped us not only to establish the individual actions the source of the initiative, but also to identify the three phases in the formation of LABS-Chennai (the ending of each is evidenced by “convergence” in the Event Structure Analysis), and abstract the micro-actions that they key actors undertook in each phase (Details in Table 2 and Figure 1 in the Discussion Section).

**Table 1:** Event Structure Analysis—Stages.

**Stage**	**Objective**	**Steps**	**Procedure in ETHNO**	**Output**
Stage 1: Converting narrative to concrete action sequence (Action principle)	Identifying from the narrative the actors, their actions and causal relations between actions	• Constructing the chronological sequence of concrete events	• Chronologically list the important events from the case narrative	Column 1 in Table 2
		2. Identifying the actors	• Identify “agents”, “object”, “setting” and “beneficiary” – attributes as defined by the ESA Analyze Program.	Columns 2–5 in Table 2
		3. Mapping causality among events/actions	• Answer a series of yes/no questions in ETHNO in order to establish the type of relations (‘historical causation’, ‘prerequisite’, ‘implications’ or ‘counterfactual relationships’) between events/actions. Iterate if needed. •Identify ‘divergences’ (events from which multiple events originate, hence the ‘cause’) and ‘convergences’ (events in which several actions converge, hence the ‘effect’) in order to bring out the causality among concrete events	Figure 1 Divergences & convergences marked in Table 2
Stage 2: Converting concrete actions to abstracted action sequence (Abstraction principle)	Creating abstractions of causal linkages from concrete events/actions	Summarizing the concrete events/actions into an abstracted event series by collapsing event networks	• Use the Summarize option in ETHNO to create abstracted event series. ETHNO finds every network of events that can be traced back to a single prerequisite, and that flows down to a single consequence, with at least one intervening event. Each of these networks constitutes a single abstracted event and is given a single event name. •Verify convergence and divergences in this stage are similar to the ones identified in the previous stage.	Column 6 in Table 2
Stage 3: Converting abstracted actions to succinct cause-effect relation (Reduction principle)	Creating a generalized model of causality	Identifying the commonality among events/actions to generalize the causal linkages between events	• Look at the abstracted events and create thematic categories (called ‘generalized events’) based on common characteristics to classify events (in a fashion similar to constructing theoretical or thematic categories in grounded theory methods Glaser and Strauss, 1967). The three event categories identified in this study are – Trigger Events, Manifestation of Personal Social Responsibility and Generative Action. •Revisit each abstracted event and decide which generalized event category it belongs to. •Answer the yes/no questions in ETNHO about the generalized events to establish the prerequisites for each generalized event. Causal linkage between generalized events represents the causality underlying the whole event series.	Columns 7-8, (in brackets)
		Verifying the causality	• Go back to the concrete events underlying each generalized event. Ask counterfactual questions about each causal linkage to see if each cause is a necessary condition to produce the effect.	

**Table 2:** Results from the Event Structure Analysis (ESA).

	**Stage 1**					**Stage 2**	**Stage 3**	
	***Column 1***	***Column 2***	***Column 3***	***Column 4***	***Column 5***	***Column 6***	***Column 7***	***Column 8***
	**Concrete events/actions**	**Agents**	**Object**	**Setting**	**Beneficiary**	**Abstracted events/actions**	**Generalized event/action**	
							**Trigger**	**Generative action**
	TQM Implementation	Executive Team	Corporation	Corporation	Stakeholders	TQM Implementation	**X**	
	Downsizing Decision	Executive Team	Redundant Employees	Plant	Stockhoders	Downsizing Decision	**X**	
Divergence 1	Acknowledging Personal Accountability for Executive Decision	PPS	Downsizing Decision	Plant	Redundant Employees, their families and communities	Social Debris Awareness		**X**
	• Look for alternate solutions •Propose union partnership •Discuss with union leadership •Convince union	PPS, Plant HR Manager	Plant HR Manager, Union Reps	Plant	Redundant workers	Forging Union Partnership		**X**
	• Talk to the Plant HR team •Map demographic and skill data	PPS, Plant HR Manager	Plant HR Team, Union Reps	Plant	Redundant Workers and their families	Demographic and Skill Mapping Plan		**X**
	• Talk to Deen about current dilemma and solutions •Learn about LABS model	PPS PPS, Deen, and DRF	PPS PPS, Plant HR Team	Chennai	Redundant workers and their families	Learn about LABS model		**X**
Convergence 1	• Identify alternate livelihood opportunities for rehabilitation of 200 families •Train family members for new jobs •Help set up entrepreneurial ventures •Help plan investment strategies	PPS, Plant HR Team, Union Reps	Redundant Workers and their families	Plant	Redundant Workers and their families and community	Successful implementation of LABS model to Plant 1		**X**
Divergence 2.1	• Reflection on success •New step to replicate in Chennai	PPS	PPS	Chennai	Chennai urban youth	Decision to replicate in Chennai	**X**	**X**
	• Talk to DRF/LABS about Chennai center •Talk to K and A	PPS, Deen	DRF team, K, A	Hyderabad, Chennai	Chennai urban youth and potential recruiters	Conversation with DRF/LABS		**X**
Divergence 2.2	• Talk to corporate executives in the network •Disagreement among corporations about ownership and conflict •Emergence of agreement around corporate consortium model	PPS, Deen, Executives from various corporation	Chennai Corporations	Chennai	Chennai urban youth and potential recruiters	Emergence of new model for LABS Chennai	**X**	**X**
	• Signing of MOU by sponsors •Form Steering Committee •A's company donates space •K's company sponsors first academy •PPS' company offers seed capital and other support •DRF recruits and trains the first set of facilitators •Advertise the program to potential recruiters and engage their PSR •Recruitment of students •Launch program with five streams	PPS, Deen, K, A, and DRF	LABS Chennai	Chennai	Chennai urban youth and potential recruiters	Formation of LABS Chennai		**X**
	• Advertise program in participating organizations seeking volunteer mentors •Train facilitators and managers in mentoring skills	Steering Committee	LABS Chennai	Chennai	LABS Chennai facilitators, and students	Developing a mentoring program		**X**
Convergenc2 / Divergence3	• Place 100% of first batch of LABS Chennai	LABS Chennai facilitators and sponsoring organizations	LABS Chennai	Chennai	LABS Chennai graduates	Successful implementation of LABS Chennai	**X**	**X**
	• Train alumni in mentoring skills and involve them in mentoring program •Move to a larger venue •Expand student recruitment to more communities •Expand more program offerings •Expand the pool of recruiters	LABS Chennai	LABS Chennai	Chennai	LABS Chennai	Ongoing expansion of LABS Chennai		**X**
Convergence 3	• DRF adopts consortium model for replication and expansion	DRF	LABS across the country	Countywide	Urban youth across the country and potential recruiters	Adoption of consortium model	**X**	**X**

The third and final phase of the analysis was undertaken for the purpose of understanding the nature and implications of the individual level micro-actions. We went over the actions undertaken by the key actors across all three stages and did a thematic grouping. We approached it in a fashion similar to constructing thematic categories or labels used in grounded theory methods (Glaser and Strauss, 1967) where similar actions are collapsed under the larger theoretical categories. We paid attention to what the actors did (the action), why they did it (the rationales offered by the actors) and what it led to (its implications for the creation of the CSR initiative) while undertaking this. This produced five categories that together represent the micro-behaviors that individuals engage in while creating CSR initiatives. Although the key actors performed actions belonging to all five categories through all three phases of developing the CSR initiative, the reliance on individual actions could be seen as decreasing in the last phase where the initiative was more or less institutionalized. In the next section, we describe the results from the iterative phases of the analysis.

## Results

### What is LABS Chennai?

Livelihood Advancement Business School (LABS) in Chennai, India is a collaborative CSR initiative of four Chennai-based corporations, viz. a manufacturing conglomorate, a consulting company, an automobile company and a food services company. LABS-Chennai focuses on skilling of underprivileged urban youth who would otherwise be cut off from opportunity and resource structures of the new economy.

Skilling is offered in areas such as auto mechanics, computer maintenance, homecare nursing, hospitality, IT-enabled services, and retail services and places them in appropriate jobs. Each area of skilling is set up as an “Academy” and a facilitator is appointed for each academy as the person-in-charge for the entire process, from student selection and training to their placement on jobs. Kulo, the LABS-Chennai Head informed: “*Everything here is systematic, in the beginning, they come in, we induct them properly and after that we give them the academic training module, work experience module and after that we place them.”* Each academy takes a proactive approach of studying the local ecosystem, comprising a market survey of the businesses and job opportunities available in the locality, and a skill-mapping of the local youth, in order to offer courses that bridge the demand and supply patterns of the local labor market.

The focus however is not on just providing the technical skills that will fetch jobs, but also on engendering life skills and career management capability that will serve the beneficiaries all through their lives. Kulo, the Head of LABS-Chennai explained: “*We do not only guide them in helping them identify the course that they can take in the next 3 months, but [also] we help them in career planning for their whole lives. What can you study? For example, if he has accounts knowledge, we recommend them to [go for higher studies beyond LABS] and study for B.Com (Bachelor degree in Commerce). We guide them throughout their careers, through their life choices.”* LABS emphasizes on an empowering approach (as opposed to philanthropic approach) as a methodology. A facilitator clarified: “*I look at empowering people through education, knowledge, generating their own values, their own resources, to help them help themselves.”* The student-centered approach aims at transformational change. A facilitator commented: “*It is a journey of self-exploration for the students and the faculty. It is a self-respect boosting journey for them. An experience focusing on the student, which is very unusual for them”*

Organizationally, LABS-Chennai is set up as a corporate consortium of the four partnering corporations, bound by a formal Memorandum of Understanding (MOU). The four corporations contributed to the seed capital and infrastructure. LABS-Chennai makes use of the skilling processes and pedagogy developed by Dr. Reddy's Foundation (DRF), a philanthropic foundation experienced in designing and implementing skilling programs across India. At the top-level, LABS-Chennai is managed by a 5-member Steering Committee consisting four executive-level representatives from each of the four corporations and one from Dr. Reddy's Foundation. All four corporations encourage their employees to participate in and contribute to the work at LABS-Chennai in various ways, viz. mentoring the students and the facilitators, providing guest lectures and organizing company visits for LABS students. These corporations also offer jobs to the LABS graduates when possible.

### Formation of LABS Chennai

A large majority of CSR models depict the CSR initiatives as a formal, rational planned process that is part of the corporate strategy. The purpose of our analysis was to uncover the individual level processes and actions that preexist and shape such initiatives. To this end, we traced the events that led to the formation of LABS Chennai. Early interviews and secondary data had revealed the involvement of four key actors—P P Sukumaran (PPS as he is generally known), Head of HR—Murugappa Group, Deen, CEO–Center for Effectiveness (a Business Process Reengineering Consulting firm), Kannan, Vice President HR—Pepsi Co, and Ajith, entrepreneur and Director–Khivraj Motors–in conceiving the initiative and making their organizations to adopt it as part of CSR. The Event Structure Analysis helped to abstract from a complex set of related events and actions, the key actions of individuals without which the initiative could not have come to existence. For brevity, we do not discuss here the findings from each stage of the analysis (Summarized in Table 2 and Figure 1). Instead, we first describe the three phases in the formation of LABS-, and then we present a conceptual categorization of the micro-behaviors that the actors were found to engage in that was revealed at the end of the analysis.

#### Phase 1: Plant level initiative

PPS, President-HR of a large Indian business conglomerate was grappling with a corporate dilemma, a downsizing decision that he needed to handle in one of the many factories of the company (referred to as the Plant in this paper). This decision was fallout of implementation of Total Quality Management system across the company. PPS was afraid that the workers may not be able to find alternate employment or fruitfully invest the money from their severance packages, and that the lack of regular income and loss of self-esteem, may turn them to take to alcoholic or other substance abuse habits.

PPS began to look for alternatives that would serve the company but without depriving 200 families of livelihood and the social impact in the small town that the factory was located. He wanted to go the extra mile that he did not need to go as the head of HR of a for-profit corporation. To begin with he talked to the Plant HR and worked on building a partnership with the workers' union. After securing the union buy-in, together, the HR team and the union mapped out the demographic details and skills sets of the families that would be affected by this downsizing and looked for helping them transition into other available livelihood options in the region.

While this was happening, PPS brought his initiative up in a conversation with Deen, a productivity consultant in Chennai and learnt that Dr Reddy's Foundation (DRF) had a skilling model that he could adopt. Therefore, using the DRF's model, PPS facilitated the rehabilitation of all the 200 families either through retraining for other employment and/or assisting them in setting up entrepreneurial ventures with their severance packages.

#### Phase 2: Deployment of the consortium-model of LABS-Chennai

Once PPS used the DRF's model to help the families of the laid-off workmen in Plant, he was so impressed with its effectiveness that he decided to replicate it in Chennai to help address the problem of urban poor being disenfranchised and disconnected from the opportunities that the new economy presented. He and Deen then reached out to several people in corporate India and Kannan and Ajith responded enthusiastically. The four then began to present the idea to a number of corporations headquartered in the city to invite them to participate in the initiative. Many of the corporations agreed that this would be a meaningful initiative; however, they were reluctant to participate in it because the model belonged to the original corporate house (DRF) that developed and deployed it. At this point there was a stalemate.

To resolve this conflict, PPS and Deen convinced DRF to consent to sharing their approach and pedagogy as one of the sponsors of LABS Chennai while allowing other local corporations to also get credit as sponsors. These organizations signed a Memorandum of Understanding to this effect. Thus, a new consortium-based model emerged in the city.

After the MOU was signed, the first step was to form the Steering Committee, which comprised of PPS, Deen, Kannan, Ajith, and a representative from DRF. This committee conducts monthly reviews of the syllabi, performance, and targets and get involved in improvement programs looking for new opportunities, new markets and new modules. They also perform the role of a boundary spanner managing the relationship with DRF. Ajith's automobile company donated space for the first center. Kannan's organization, a food services company, sponsored the first academy, which consisted of 35 students who would then be absorbed by the company on their graduation from LABS. PPS's organization offered seed capital and other network related resources to the center. DRF/LABS set up the training of the facilitators and the transfer of knowledge and skills.

Then, the facilitators and DRF managers supported by the steering committee began recruitment of students from underprivileged urban communities. Steering Committee members took the lead in advertising the program to potential employers in their professional networks. The first batch of LABS Chennai began with five streams/academies. Next, the Steering Committee members advertised in their organizations seeking managers who would volunteer to be mentors to the students as well as facilitators. Thus, the mentorship program began. Training of the first batch was completed and resulted in 100% placement.

#### Phase 3: Institutionalization of the consortium model of LABS-Chennai

This success led to further expansion of the mentoring program to include alumni as mentors to the subsequent batches of students. Alumni and current students were also involved in recruiting new students from their communities. LABS-Chennai expanded the number of program offerings to eight and also moved to a larger venue. By this time, the center was financially self-sustainable with the recruiting fee or sponsorship provided by the recruiters. They also expanded the pool of recruiters.

Success of this consortium-led model inspired Dr Reddy's Foundation to adopt the same while opening similar skilling centers in other cities. This meant a shift for Dr Reddy's Foundation from the traditional philanthropic model that relied on the donations from the benefactors to set up the centers, to a co-ownership model that elicited greater involvement from the partnering corporations in the strategic as well as operational aspects of running the centers. This was felt more beneficial in bridging the gap between the underprivileged and the Corporate India.

### Conceptual categorization of the micro-behaviors of individuals creating CSR initiatives

The later stages of Event Structure Analysis enabled unpicking the key actions of individuals without which the initiative could not have been created (For details, see Stages 2 and 3 in Table 1). A thematic grouping of these key actions yielded five conceptual categories that summarize the micro-behaviors that the individuals engage in to create CSR initiatives (Table 3). These included *increasing stakeholder salience by turning attention to the ethical and social responsibilities to specific stakeholder groups, emerging as a self-appointed CSR champion by assuming personal responsibility for action, creating CSR initiative prototypes by leveraging personal skills, garnering support by leveraging personal networks* and *amassing operational resources by leveraging organizational resources*. These micro-behaviors unfolded not necessarily in a sequence, but often in a parallel and mutually reinforcing manner.

**Table 3:** Thematic Categorization of Micro-actions of Individuals.

	**Key events/actions**	**Sub events/Actions**	**Actors**	**Micro-action categories**				
				**Turning attention to specific stakeholders**	**Assuming personal responsibility**	**CSR solution via Personal skills**	**Garnering support via personal Networks**	**Operational resources via organizational resources**
Phase 1	Downsizing	TQM implementation & downsizing decision	Org. actors: Executive Team					
	Forging Union Partnership	Look for alternate solutions	Ind. actor: PPS Org.actor: Plant HR Manager	**X**	**X**			
		Propose union partnership						**X**
		Discuss with union leadership						**X**
		Convince union				**X**		
	Demographic and Skill Mapping Plan	Talk to the Plant HR team	Ind. Actor: PPS Org.actor: Plant HR Manager			**X**		
		Map demographic and skill data						**X**
	Learn about LABS model	Talk to Deen about current dilemma and solutions	Ind. Actors: PPS, Deen Org.actor: DRF				**X**	
		Learn about LABS model						
	Successful implementation of LABS model to Plant	Identify alternate livelihood opportunities for rehabilitation of 200 families	Ind. actor: PPS Org.actor: Plant HR Team, Union Reps			**X**		
		Train family members for new jobs				**X**		**X**
		Help set up entrepreneurial ventures				**X**		**X**
		Help plan investment strategies				**X**		**X**
Phase 2	Decision to replicate in Chennai	Reflection on success	Ind. actor: PPS	**X**				
		New step to replicate in Chennai			**X**			
	Conversation with DRF/LABS	Talk to DRF/LABS about Chennai center	Ind. actors: PPS, Deen			**X**		
		Talk to K and A					**X**	
	Emergence of new model for LABS Chennai	Talk to corporate executives in the network	Ind. actors: PPS, Deen, K, A Org.actors: Executives from various corporation				**X**	
		Disagreement among corporations about ownership and conflict		**X**				
		Emergence of agreement around corporate consortium model			**X**	**X**		
	Formation of LABS Chennai	Signing of MOU by sponsors	Ind. actors: PPS, Deen, K, A Org.actor: DRF					**X**
		Form Steering Committee				**X**		
		A's company donates space						**X**
		K's company sponsors first academy						**X**
		PPS' company offers seed capital and other support						**X**
		DRF recruits and trains the first set of facilitators						**X**
		Advertise the program to potential recruiters and engage their PSR						**X**
		Recruitment of students						**X**
		Launch program with five streams						**X**
	Developing a mentoring program	Advertise program in participating organizations seeking volunteer mentors	Ind.actors (formal role): Steering Committee					**X**
		Train facilitators and managers in mentoring skills				**X**		
	Successful implementation of LABS Chennai	Place 100% of first batch of LABS Chennai	Org.actors: LABS Chennai facilitators and sponsoring organizations				**X**	**X**
Phase 3	Ongoing expansion of LABS Chennai	Train alumni in mentoring skills and involve them in mentoring program	Ind.actors (formal role): Steering Committee Org. actors: LABS Chennai, Consotium			**X**		**X**
		Move to a larger venue						**X**
		Expand student recruitment to more communities						**X**
		Expand more program offerings						**X**
		Expand the pool of recruiters					**X**	**X**
	Adoption of consortium model	DRF adopts consortium model for replication and expansion	Org. actor: DRF					**X**

#### Increasing stakeholder salience by turning attention to the social and ethical responsibilities to specific stakeholder groups

This involves actors developing a heightened sense of the social and ethical responsibilities to specific stakeholder groups in situations that others perceive as “business as usual.” In this case, none of the individual actors held formal CSR roles in their organizations, or were under formal contractual or compliance oriented obligations or unwritten expectations on the part of their organizations to consider stakeholder interests beyond what their job roles required. However, they picked upon triggers from both the organizational and social environments at the macro level and emotional and moral factors at the micro level to reflect upon the wider social responsibilities that the situation called for.

In the first phase, PPS recognized the probable societal level negative consequences of his employer's decision to downsize on the workers and their families. Workers and families were an easily identifiable stakeholder group for PPS' organization, but organizations usually focus on their legal responsibilities toward the workers they make redundant, especially if financial concerns drive layoffs. However, PPS wanted to make sure “*that we don't leave behind debris in that sense we don't actually leave behind people who will actually become a social burden.”* His take on the situation turned the organization's attention to its social responsibility to this stakeholder group.

Similarly, in phase 2, the key actors, PPS, Deen, Kannan and Ajith draw attention to the social responsibilities that their organizations have to the underprivileged urban youth in Chennai. All their organizations are located in Chennai, an Indian city that has attracted a large number of big corporations to set up and expand their operations there. Although these corporations have created jobs, they are largely accessible to the middle class, and this has created widening inequalities between the rich and the poor. The underprivileged urban youth with little prospects of landing on a well-paying job are at the risk of turning to the darker sides of the economy for a living. Kannan pondered, “*If you were to find out some of the boys have become pick pockets, or gotten into drugs or in any such negative spirals, I don't think it is because of their interest, or because they want to be that way. It is largely their circumstances and lack of mentoring and guiding them. There are many such in the society. Somewhere I think, we have a role to play.”* By repeatedly emphasizing these groups as legitimate stakeholders that their organizations should pay attention to, the key factors contributed to increasing the salience of this stakeholder group for their respective organizations, and compelled them to rethink organizations' social and ethical responsibility to this stakeholder group.

As the shared understanding of the salience of this stakeholder group increases within the organizational contexts, the actors might feel lesser need for advocacy work. In phase 3, the key actors were seen doing less of advocacy work within their organizations, but directing it to the external audience when they do. They leave it for Dr Reddy's Foundation to spread the message to corporations located in other cities.

#### Emerging as a self-appointed CSR champion by assuming personal responsibility for action

The individual actors were found to take personal responsibility for action, rather than leaving action to their organizations. This stemmed from a sense of personal accountability to the stakeholders originating from a sense of personal connection to the context and stakeholders.

Many executives who deal with layoffs feel no obligation to help laid off workers or their families and communities; for them downsizing decisions are just a part of doing business. However, reminiscing about phase 1, PPS shared that he felt “*there is something called the emotional accountability,”* and as “*someone who is attached to it*,” he could not just walk away. This prompted him to get into action instead of leaving the job to the HR Head at the Plant.

All key actors involved in the second phase, variously expressed the sense of connection and responsibility they felt toward underprivileged urban youth. Kannan said: “*These boys are very true to themselves, they have no ulterior motives, right? They only want to have a good living, a very sincere living, they want someone to advise them. Where things go wrong is, when there is nobody to advise them, mentor them, things go wrong, you know.…[so] we have a role to play. If we can play whatever small role to help them, it makes a lot of difference.”* As a result of this, they engaged in substantial amounts of informal and *ad-hoc* activities and got their hands dirty, before their organizations got on board. This gave them visibility as informal CSR champions within their organizations, and led them to be placed in the more formal roles such as the Steering Committee Member once the initiative was formalized.

Once formalized, the nature of personal responsibility for action was seen to change. In phase 3, while all four key actors continued their association with the initiative, their engagement was more bound by the role (as the Steering Committee member), where as it was more organic and emergent in the previous phases. PPS said: “*[As Steering Committee, W]e generate ideas, and inspire people within the board, Deen, myself, the Regional Coordinator, and we also meet with Dr Reddy's Foundation once in a quarter to see if they have any new ideas so we can learn from them, and if we have new ideas they learn from us…. We also look at resources. We are also planning a visioning exercise for a long-term plan which is like a typical business plan for LABS*.”

#### Creating CSR initiative prototypes by leveraging personal skills

The key actors leveraged their leadership and strategic skills to create solutions to the issues facing the stakeholders and formalizing them into a CSR initiative. In professional capacities, two of the actors were heads of HR of two big corporations, one an organizational consultant and the last an entrepreneur. Their professional training and experience reflect in the solutions they generated. Their efforts, in a way, made readily implementable CSR solutions available to their organizations. It could be also seen that the individual-driven solutions might not fully satisfy the more formal and strategic concerns of the organizations. However, the willingness of the individuals to continue applying their problem solving skills helped these emergent solutions to evolve further and reach a form that was acceptable to the organizations.

In phase 1, faced with the issue of livelihood for the soon-to-be redundant workers, PPS drew on his long term HR expertise and devised a solution involving skill mapping and reskilling. Testing the solution at the plant enabled him to develop it into a replicable model for setting up a similar initiative in Chennai. This served as a prototype in the key actors' discussions with their organizations, which helped to convey the level of commitment and investment needed on their part. This helped to get an acknowledgment from the corporations that it was a worthy cause for CSR. However, they were not ready to make the commitment because of the strategic and ownership concerns. Deen shared: “*It was a conflict that came up when we started making presentations to other corporates, we could feel the need; they said, if it is [the process model from] Dr. Reddy's Foundation, why don't you get all the funds from them? If we want to participate, can this be an open forum?”*

Therefore, much of the early work in Phase 2 for the key actors was to come up with an organization model for LABS-Chennai that would alleviate the corporate concerns about ownership and claiming of credit for CSR work. PPS talked about the design issue they faced: “*I think the name and the style of the organization and the financial structure is very important. So we are trying to put together a structure but which this is an open forum where people can come together and participate without the limelight being hogged by XYZ, in terms of people or organizations.”* The actors leveraged their entrepreneurship and consultancy expertise to come up with the consortium-model. Deen informed: “*So today we have actually formed some kind of corporate consortium and we have signed a MOU on a long term basis, so today it doesn't belong to anyone, at the same time it belongs to five corporates.”*

In phase 3, the key actors were not seen as developing radically new solutions, but mostly employing their leadership skills in maintaining the model that was now institutionalized. While they provided strategic leadership to LABS-Chennai, they were seen to visit LABS centers in other cities to interact with facilitators and students, mainly to inspire them. Concurrently, the task of disseminating the model as a replicable CSR model to other corporations was left to Dr Reddy's Foundation. Bala, a manager from Dr Reddy's Foundation clarified: “*…we replicate our success. We will be helping them [Corporations] run the initiatives. Like the general MOUs any corporation has. We have set teams who handle these processes. We also have a fairly set process. The process starts with a market scan, we identify the local talent, skills, jobs. Initially, I remember that we didn't know how to do it. Now we have a well-established process, that too, in a set number of days, set number of people, what kind of people should be in the team–Around 10 days to do the market scan, another 5 days to recruit and select the candidates, another 5 days for curriculum development, which happens simultaneously, and then the capacity building activity happens, the actual training. These are the costs involved with these. We tell them [what the cost is] and it will be directly paid to us. We are a non-profit and so we only charge costs.”*

#### Garnering support by leveraging personal networks

The key actors sought to garner support via their personal networks and connections. It is noteworthy that oftentimes they turned to connections outside of the organizational boundaries. This helped gathering fruitful ideas to test and form a coalition of the like-minded to spearhead the initiative.

In Phase 1, when PPS was contemplating an intervention at the plant, his source of information and support was Deen, someone with whom he had professional and personal relationship, but belonged to another organization. The DRF connection that helped PPS for rolling out the program at the plant was Dr. Nalini Gangadharan, Deen's classmate from graduate school.

In Phase 2, Deen managed to rope in Kannan and Ajith, who were both Deen's clients from two other organizations. Kannan remembered: *If Deen hadn't come and told me about LABS, I wouldn't have known about it. I was introduced to LABS through Deen, Deen as a consultant, whoever he meets up as a client, he makes it a point to talk about LABS during the last 10 min after the business is over. He did talk to me and it really touched me.'* The coalition that PPS, Deen, Kannan and Ajith formed stayed as the central pillar holding up LABS-Chennai in the years to come. Together, they made presentations to their own as well as other organizations seeking support for the formation of LABS-Chennai. Once LABS-Chennai was formed, they continued to call in on their contacts for professional help and advice. “*Absolutely, [we call on] not only corporations but also our personal networks among corporations to get people with know-how. For example, if we are having a problem in the quality of programs, then we get someone from IIIC [Training Center of the company] to come and evaluate it and give suggestions, it may be anyone…. But the sheer network, I ring up GP to send Joseph to see how the quality of programs is, can you develop measures of performance to see if they programs are going well, as an outsider what do you see?”* As LABS-Chennai matured, volunteer mentors and paid facilitators also were recruited through personal networks and professional interactions.

In phase 3, the key actors continued to spread the message about LABS among the new contacts they made, which served as PR for the initiative. Deen opined: “*There is no point in talking to the whole world about it. Instead, do it in a small way wherever we can. I do it in my business model, I do it with LABS. I don't think we can do it in a big way. Though I try, in my meeting with Abdul Kalam (then President of India), or whoever I meet, I spend the final 15 min of my presentations on this. In some places, it works, in some places, it doesn't.'* In the meanwhile, there was greater emphasis on building LABS-Chennai's own networks for ensuring continued people support for its activities. One key network formed was the alumni network of those who have graduated from the programs. Alumni volunteered as mentors to the succeeding cohorts of students. The vision is “*to get the alumni involved, if the alumni came in slowly as faculty, alumni can evolve programs, alumni can do the market research, we are trying to do that slowly*.” This indicated a shift in the nature of network engagement as the initiative is institutionalized.

#### Amassing operational resources by leveraging organizational resources

In addition to their skills and networks, the key actors also made use of their formal organizational roles to gather resources from within their organizations for implementing the initiative. This included human, financial and infrastructural resources. Occupying roles at the top levels of the organization might have made it easier for these actors to access such resources, although they also had their share of barriers.

In the phase 1, PPS as Head-HR was able to get the help of the Plant HR head in interfacing with the union, and from the union to plan and implement the initiative at the plant level. A higher position in the organizational hierarchy seemed to have helped in enlisting such help.

In phase 2, the key actors were in need of more resources for setting up the lab formally. Their organizational roles made it easier to get audience with the key decision makers in their respective organizations. Although the initial responses were not entirely favorable, their positions allowed them to keep the conversation channels open and check if the solutions they devised were acceptable to the other actors in decision making roles. This allowed them to secure the financial and infrastructural resources to start the LABS-Chennai center. Further, they used their organizational roles to seek the involvement of their peers and subordinates in the operational aspects of running the center, thus encouraging the transfer of corporate know-how to LABS-Chennai. Kannan commented, “*The way I see it, what we…are doing is really technology transfer; we have learnt some skills as a business, like strategy, operations, sales, reviews and process management and we are transferring this technology to a non-profit venture*.”. This helped in particular to get a number of executives to volunteer as mentors to the facilitators and students at LABS-Chennai.

In phase 3, as the initiative was institutionalized, there was a far greater need for organizational resources as reliance on key actors' personal skills and networks decreased. One key effort was to enable LABS generate its own financial resources and achieve financial self-sustainability as an organization. This was done through charging a fee to organizations for recruiting labs graduates or a price for offering customized skilling programs. At the same it was felt that involvement of more corporations from different sectors is still a necessity. Rajesh, a LABS facilitator informed: “*The main process that runs this is the corporate support like Murugappa Group, Rane Group, Khivraj who are supporting this venture. How many ever ideas we have, we need the resources to implement it. We get funds from all these corporations. They are getting involved in a not-for-profit mode, this venture is not to add to their wealth, which is great. Corporations like this get involved in livelihoods, may be they feel that they have earned a lot and they want to do something back for the society.”*

## Discussion and conclusions

Although the potential of individual stakeholder behaviors to shape organizational outcomes in CSR is widely acknowledged (Hemingway and Maclagan, 2004; Porter and Kramer, 2011; Amit and Zott, 2012; Littlewood, 2014), empirical literature on the individual actions and behaviors that influence and shape organizational CSR is still emergent (Hemingway, 2005; Aguinis and Glavas, 2012; Griffin and Prakash, 2014; Constantinescu and Kaptein, 2015; Ghobadian et al., 2015). This may be due to the overwhelming focus of the past CSR research that examines the relationships between micro and macro levels, on the effect of organizational CSR on employees, but not on the behaviors of individual employees within organizations who act as corporate entrepreneurs and drive and shape CSR initiatives of their companies. In this study, we set out to empirically explore the micro actions influence and shape organizational CSR. Based on our case study findings, we make two contributions to the literature: First, we offer a categorization of individual micro-behaviors that contribute toward the development of organizational CSR initiatives. Second, we explicate the characteristics of individual approach to CSR that makes it different from, but complementary to organizational approach to CSR.

### Individual micro-behaviors shaping organizational CSR

This study revealed five categories of micro-behaviors, namely *increasing stakeholder salience by turning attention to the ethical and social responsibilities to specific stakeholder groups, emerging as a self-appointed CSR champion by assuming personal responsibility for action, creating CSR initiative prototypes by leveraging personal skills, garnering support by leveraging personal networks* and *amassing operational resources by leveraging organizational resources*.

#### Increasing stakeholder salience by turning attention to the ethical and social responsibilities to specific stakeholder groups

Stakeholder salience has been identified as one of the major factors that influence an organization's ethical and responsible actions to various stakeholder groups (Jones et al., 2007). Key proponents of the concept of stakeholder salience argue that organizations find it difficult to satisfy the mutually competing interests of various stakeholder groups and the degree to which organizations give priority to any particular group depends on the relative power, legitimacy, urgency and/or proximity of a group over the others. Managers are actors who interpret on their organization's behalf whether a particular group's interest is legitimate or worth immediate attention. Managers' proximity to the groups also might make a difference how they choose to interpret the organization's ethical or social responsibility to specific groups. The key actors in this case turn their attention to stakeholder groups that their organizations attach lesser or no salience. By acknowledging the issues faced by these groups and articulating how these issues should be a concern for their organizations, they legitimate the interests of the stakeholder groups and establish them as those that require urgent organizational attention and intervention.

#### Emerging as a self-appointed CSR champion by assuming personal responsibility for action

Individual actors who assume personal responsibility are likely to act as self-appointed CSR champions (Hemingway, 2005). They might be more vocal and actively involved in shaping organizational CSR. The chances are these actors will be more passionate about the CSR initiatives and putting more energy in making them work, than actors with formal CSR roles. Their actions could be informal and hands-on yet highly visible to other members of the organization, thus influential in developing an organizational environment favorable to CSR (Drumwright, 1994).

According to Hemingway and Maclagan (2004) what differentiates those who act from those who do not is taking personal responsibility for action. People who act place the locus of responsibility in themselves, while those who refrain place it on the organization.

Takkala and Pallab (2000, p. 112) also suggest that individuals who assume *a duty or obligation* act *voluntarily* without being required by their formal job roles, *willingly* with “full cognizance of relevant circumstances and personal repercussions,” and *intentionally* with focus on outcomes. According to Hemingway (2005), “championing of CSR depends upon a salient sense of personal responsibility” (p. 237). This study suggests that the propensity to assume personal responsibility varies from individual to individual. Not every executive who have had to with layoffs thinks about the impact on families and community and feels the moral responsibility to salvage the livelihoods of laid off workers. Even if they feel cognitive dissonance, they may be able to come to terms with it by framing it to themselves as unavoidable business decisions. The case supports DeCelles et al.'s (2012) finding that powerful individuals with a stronger sense of moral identity tend to act less in self-interest. *Assuming personal responsibility for action leads to championing CSR at organizational level*.

#### Creating CSR initiative prototypes by leveraging personal skills

The case shows that the initial CSR solutions were developed by individual actors who leveraged their personal leadership and strategic skills for the purpose. Given that many individuals do not seek their organization's approval or support when they start the initiatives (Drumwright, 1994), it is only natural that they rely on their own personal resources. Individual attempts however makes readily implementable CSR solutions available to their organizations. The emergent and evolving nature of solutions individuals offered might make them more flexible than rational organizational solutions. Smaller scale testing of the solutions by individuals enables creation of replicable models. This actually allows organizations to see a prototype of the solution in action and get an idea about the level of commitment and investment needed on their part. In the event that it does not generate enough interest from the organizations, the individual actors tweak the solutions in ways that it can work with the level of resources that the organizations are willing to commit. This allows the organizations to optimize their levels of involvement. Individual actors were involved in the ongoing monitoring of the initiative, making it easier for their organizations to keep track of the progress and changes required. When individuals thus lead CSR thus, it lowers the risks of organizational CSR, not least because any failure that occurred wouldn't be seen as the failure of organizational CSR.

However, the effort, expertise and resources needed for many CSR initiatives may be more than what a single individual can offer. Research shows that individuals find it easier to engage in CSR when they find support within their organizational environment, from supervisors (Ramus and Steger, 2017) and corporate practices (Den Nieuwenboer and Kaptein, 2008). Those who do not find support or resources in their immediate environment may either become frustrated and abandon action (Hemingway, 2005) or seek to find it through their networks outside. When seeking support through networks within or outside the organizational boundaries, the individuals are also spreading CSR awareness and getting more people involved. This is crucial in transforming the personal initiatives to collective initiatives.

#### Garnering support by leveraging personal networks

Leveraging personal networks helps to generate ideas as well as form partnership with kindred spirits in the early phases of developing a solution. In the later phases of implementation and institutionalization, it contributes toward building awareness about CSR in general and specific initiatives in particular in the respective organizations. The networks of the individual actors may include members of the top management, their peers and subordinates in the organization. In their conversations with members at different levels, they consciously attempt to build awareness and amass support. While this may prompt top management to make decisions favorable to CSR, it also encourages other actors to take actions in support of CSR. These individuals may actively seek to spread the message through their networks outside the organizations as well. This serves as active PR for the initiative. The participating organizations also benefit from this PR as they present them as legitimate CSR actors. Thus, breaching the organizational boundaries by individual actors serves the interest of organizational CSR.

#### Amassing operational resources by leveraging organizational resources

Leveraging of organizational resources is an essential action as it differentiates CSR activities of individuals from philanthropic and volunteering activities they undertake in personal capacity. Unlike personal philanthropy and volunteering, where individuals expend only their personal time, wealth and skills, individual-level CSR involves their reliance on human, monetary, and infrastructural resources available to them through their ties to an organization.

This action is also vital in institutionalizing CSR initiatives and reducing the dependence on single individuals for its sustenance. The actors in this case occupied fairly senior positions in their organizations, and had positional power and legitimacy. This probably made it easier for them to influence decision-making processes (Agle and Caldwell, 1999) and resource allocation (Augilera et al., 2007).

### Differences and complementarities between individual and organization-driven CSR

This study also reveals several key differences between individual and organization-driven CSR, which also become the sources of complementarities. Firstly individual-driven CSR is, most likely, more stakeholder or cause oriented, whereas organization-driven CSR tends to more business-case oriented. Although in the end, it resulted in building a good business case. Organization-driven CSR, with its utilitarian approach to building a business case for CSR, has made organizations neglect the social aspect of CSR and ignore the real CSR causes, where radical transformation is necessary (Augilera et al., 2007). Individuals who choose to start socially responsible actions on their own might be normatively moved primarily by the plight of potential beneficiaries (e.g., redundant workers, underprivileged urban youth) or the cause itself (e.g., clean energy), rather than a motive to help their organization to improve its image. As a result, individual-driven initiatives may be more transformation-oriented compared to organization-driven initiatives that are more likely to be short-term and superficial public relations and image management exercises.

Secondly, the locus of responsibility is different in individually driven CSR and organizationally driven CSR (Hemingway and Maclagan, 2004). A sense of personal responsibility—when individual actors take personal responsibility to act in situations in which they find their organizations could be socially responsible—drives individual driven CSR (Hemingway, 2005). Organizationally-driven CSR relies more on role-related responsibility than personal or individual responsibility. Individual actors in organization-driven CSR are merely representatives of the organization discharging their responsibility on behalf of the organization.

Thirdly, individual-driven CSR may be more emergent as it is rare that individual managers start their jobs in for-profit firms with CSR in mind, unless they are hired for CSR roles (Pedersen, 2010). Most managers have a “do no harm,” rather than a “do good” attitude toward social responsibility, and are usually less concerned about stakeholders other than employees and customers (Ibid). When managers decide to take positive actions, it is usually in response to a trigger event, which makes them feel that they or their organizations are responsible toward a certain group of stakeholders or a cause. The actions therefore may not be pre-planned, but spontaneous and evolving as the actions themselves unfold. On the other hand, organization-driven CSR is likely to be more planned as most organizations now approach CSR with a strategic intent, thanks to the prevalent business case rhetoric (Carroll and Shabaana, 2010). In the current scenario, organizations feel the need to present themselves as socially responsible actors and hence are likely to use CSR as part of corporate strategy and planning (e.g., use of Fair Trade labels).

Fourthly, individual-driven CSR may not always take place under the corporate CSR banner, even if the individuals use their organizational roles and resources for such initiatives. Past literature reports instances where managers champion initiatives based on their personal beliefs (Fineman and Clark, 1996). Individual initiatives could run parallel to or separate from organizational initiatives. The organization may or may formally recognize their intiatives. In some cases, they could be merged into organizational initiatives after an initial period of independent running. This might depend on if the organization finds it a worthwhile cause to be part of the formal CSR program, and the costs and benefits involved.

Finally, individual-driven CSR need not confine itself to a single organization and can benefit from connecting multiple organizations and networks. As seen in this case, the personal networks of individuals pioneering such initiatives span organizational boundaries and as a result they might draw in resources from external sources. Organizational CSR focused on business case may be more centered on a single organization. One reason could be the business case driven approach (Carroll and Shabaana, 2010), which is concerned with getting the maximum mileage for the organization and hence has a reluctance to partner with other organizations and share the glory. The other reason could be the superficial nature of initiatives—the organization may not be willing to get involved in any radical social transformation initiative (Augilera et al., 2007) that requires collaboration with multiple partners. Public-Private partnerships cultivated by international companies such as Unilever are focused on capitalizing on the bottom of the pyramid by expanding the consumer base and not aimed at radically changing social norms (Poonamallee, 2011). Despite these differences, both individual and corporate moral agency can influence and shape each other (Constantinescu and Kaptein, 2015).

### Implications for practice and research

While findings from a single base study may not be broadly transferable, our case suggests that the mix of different kinds of micro-actions that drive a CSR agent, is emergent and iterative, and might go through several phases. The frequency of each type of action might vary with each new phase. If the individuals cease to assume personal responsibility for further action at any point, the process could stop. Alternatively, institutionalization might make personal responsibility less necessary. In this scenario, locus of responsibility would be shifted to individuals who are assigned with formal CSR responsibility within the organizations. In case it remains as individual-driven CSR, the chances are that individuals will have to keep engaging in all the micro-actions all the way through. But if the plan is to institutionalize the initiative, there will be greater leveraging of the organizational resources in the later stages and lesser reliance on individual's personal resources and networks. Understanding and explicating these boundary conditions through empirical research will be useful addition to multi-level CSR practice and research.

A limitation of this study is that it is guilty of looking at this initiative from the perspective of top management members who hold positional power and access to organizational resources. But research suggests that these individuals could be from any level in the organization (Hemingway, 2005), and middle and lower level managers can set the moral tone of an organization (Drumwright, 1994; Marz et al., 2003). In fact, people with higher status in terms of power and income are less likely feel empathy and act in less prosocial ways (Galinsky et al., 2006). While this study confirms DeCelles et al.'s (2012) finding that power when coupled with a strong moral identity can lead to more prosocial behaviors, we also urge further research into understanding where more bottom-up CSR initiatives led by lower and middle level managers and workers fit in the integrative CSR model. In the absence of comparative studies of individual actors at different levels that examines similarities and differences in their motives, actions and outcomes generated, it is hard to say conclusively to what extent organizational roles and power affect an individual's ability to be effective CSR actors. It remains a question whether managers or employees at middle or junior levels find it equally impactful or if lack of power or legitimacy may force certain actors to be more covert or even subversive in taking their CSR agendas forward (Hemingway, 2005). Understanding this dynamic will help organizations in fostering and supporting individual CSR initiatives.

## Ethics statement

The proposal was reviewed by Case Western Reserve University and Institutional Review Board. Participation was voluntary and non-invasive. Participants were informed of confidentiality but several of the key players chose to forgo anonymity. They all signed an informed consent form.

## Author contributions

LP collected the data and led the analysis and SJ helped in theorizing and deepening the analysis.

### Conflict of interest statement

The authors declare that the research was conducted in the absence of any commercial or financial relationships that could be construed as a potential conflict of interest.
